# The Comparative Effectiveness of Intravenous Immunoglobulin and Corticosteroids in Kawasaki Disease: A Nationwide Claim Data Analysis

**DOI:** 10.3390/jcm14062012

**Published:** 2025-03-16

**Authors:** Sujin Lee, Seeun Choi, Kiyon Rhew

**Affiliations:** 1 College of Pharmacy, Dongduk Women’s University, Seoul 02748, Republic of Korea; 30805@snuh.org (S.L.); 20244093@dongduk.ac.kr (S.C.); 2 Department of Pharmacy, Seoul National University Hospital, Seoul 03080, Republic of Korea

**Keywords:** Kawasaki disease, immunoglobulin, corticosteroid, pediatric, vasculitis

## Abstract

**Background**: Kawasaki disease (KD) is an acute type of vasculitis in children, with coronary artery aneurysm (CAA) being its most serious complication. Intravenous immunoglobulin (IVIg) with acetylsalicylic acid (ASA) is the standard treatment, but concerns about IVIg’s availability and adverse effects have led to interest in corticosteroids (CSs) as an alternative. This study compares the clinical outcomes of IVIg and CSs in KD patients. **Methods**: Using South Korean Health Insurance Review and Assessment Service (HIRA) data from 2017 and 2020, we identified children under five diagnosed with KD and treated with IVIg and ASA (the IVIg group) or CSs and ASA (the CS group). Propensity score weighting was applied. The primary outcome was the incidence of CAA, and the secondary outcome included cardiovascular complications. Cox proportional hazards models were used to estimate the hazard ratios (HRs) with 95% confidence intervals (CIs). **Results**: After adjustment, the CAA incidence was higher in the CS group (6.72%) than in the IVIg group (1.86%) (HR = 3.70; 95% CI: 1.96–6.97; *p* < 0.0001). Cardiovascular complications were also more frequent in the CS group (HR = 2.87; 95% CI: 1.96–6.97; *p* < 0.0001). **Conclusions**: The combination treatment, of CSs and ASA, was associated with a higher risk of CAA and cardiovascular complications compared to that when using IVIg in pediatric KD patients. While CSs may be beneficial as an adjunct therapy in IVIg-resistant cases, they should not replace IVIg as a first-line treatment. Alternative strategies, such as reduced-dose IVIg with adjunctive therapies, should be explored.

## 1. Introduction

Kawasaki disease (KD) is an acute febrile type of vasculitis that primarily affects children under five years old and is typically a self-limited disease [1]. However, some patients develop necrotizing arteritis during the acute phase, leading to arterial wall damage and cardiovascular complications, most notably coronary artery aneurysms (CAAs), the most common and serious complication of KD [1,2]. Consequently, the prognosis of KD depends on cardiovascular complications such as CAA, cardiovascular collapse, myocardial dysfunction, valvular and aortic abnormalities, and arrhythmia. These complications increase morbidity and mortality. Intravenous immunoglobulin (IVIg) and high-dose acetylsalicylic acid (ASA), unless contraindicated, have been established as the standard treatment for KD in the acute phase, as multiple studies have demonstrated their effectiveness in reducing the incidence of CAAs [3,4,5]. A meta-analysis showed that the use of high-dose IVIg and high-dose ASA in the initial treatment of KD reduced the prevalence of CAAs from 15 to 25% and to nearly 4% compared to ASA alone and low-dose IVIg [6].

Although IVIg is highly effective in reducing the prevalence of CAAs, there are several limitations to the administration of IVIg to young children. First, there may be important manufacturing differences, and although many studies have evaluated the therapeutic effects of IVIg from different manufacturers, their results are conflicting [7,8,9]. Additionally, IVIg can cause Coombs-positive hemolytic anemia or aseptic meningitis, making it contraindicated for some patients [10,11,12]. Also, since KD most frequently affects children under five, IVIg therapy can also impact the immunization schedule [13]. Lastly, instability in the IVIg supply can also be a significant obstacle to IVIg therapy [14,15]. Over the past 20 years, the use of IVIg has more than tripled due to advancements in the diagnostic techniques and expanded indications. However, problems often arise with the global supply of IVIg, leading to disruptions in the raw material supply chain [16,17].

Corticosteroids (CSs) were used to treat Kawasaki disease before IVIg was used and are still used with IVIg and ASA as an adjuvant therapy in patients with resistance to IVIg therapy or as initial combination therapy in high-risk patients [18]. Additionally, a recent study showed that the incidence of coronary artery lesion (CAL) complications was not statistically different when comparing a group using CSs and a group administered IVIg [19]. In addition, in terms of the drug use in patients with Kawasaki disease in Korea, it was reported that in patients (113 patients) who did not take IVIg, there were no cases during the 3-month follow-up period and just one case during the 6-month follow-up period [20]. In addition, a recently conducted meta-analysis reported that when IVIg treatment was compared with prednisolone as the first-line treatment in KD patients, the incidence of CAAs (odds ratio: 0.60; 95% confidence interval (CI): 0.24 to 1.48) was not statistically significant, and the number of studies was not sufficient, so there was insufficient evidence [21].

CSs are used for IgA vasculitis or chronic vasculitis in children [22,23]. Steroids effectively treat joint and gastrointestinal symptoms in patients with IgA vasculitis who do not respond to supportive care. In addition, the treatment with the most established evidence for children with other types of chronic systemic vasculitis is CSs [24,25]. Steroids generally reduce inflammation by inhibiting the synthesis of cytokines, which cause inflammatory responses [26]. Therefore, we sought to evaluate whether CSs could replace IVIg by comparing the use of IVIg with the use of CSs in Kawasaki patients. While IVIg remains the standard treatment for KD, concerns regarding its availability, potential adverse effects, and impact on immunization schedules have led to interest in alternative therapeutic options.

Given the limited number of studies directly comparing IVIg and CSs as first-line treatment options, further investigation is needed. This study aims to explore the clinical outcomes and evaluate whether CSs could provide a similar benefit with fewer limitations in certain subsets of KD patients, ultimately broadening the treatment options. Rather than proposing CSs as a definitive replacement, this study seeks to contribute to understanding their potential role in KD management and to assess whether they could serve as a feasible alternative in specific clinical scenarios where the use of IVIg may be challenging.

## 2. Materials and Methods

### 2.1. Study Subjects

This study used pediatric sample data, randomly extracting a stratified 10% sample of pediatric patients (~1,100,000) under 20 years old who accessed medical services within a given year from pediatric and adolescent patients from 2016 to 2020. However, the Health Insurance Review and Assessment Service (HIRA) information was masked due to privacy protection policies for rare diseases. KD was categorized into specific years (2016, 2018, and 2019); the analysis was limited to the unmasked years, 2017 and 2020, from the 2016–2020 dataset (HIRA-PPS-2017, HIRA-PPS-2020). National Health Insurance (NHI) is South Korea’s public health insurance system, covering most of the population [27]. All medical activities are reported to HIRA, which collects and analyzes data from medical institutions. This study included children under 5 years of age diagnosed with KD and administered with high-dose IVIg (2 g/kg/day for 1 day via IV infusion) and ASA (the IVIg group) or corticosteroids and ASA (the CS group). The exclusion criteria were children who received IVIg and CSs simultaneously or consecutively (even if they were administered at different time intervals). Patients who had been diagnosed with the outcomes evaluated in this study, including CAA, before being diagnosed with KD were also excluded. The ATC (Anatomical Therapeutic Chemical) codes of the drugs used in the analysis were ASA (acetylsalicylic acid, B01AC06, N02BA01), IVIg (J06BA02), and CSs (methylprednisolone, H02AB04; prednisolone, H02AB06).

### 2.2. Definitions of the Disease and Study Outcomes

KD and cardiovascular complications and other diseases were defined using the Korean Standard Classification of Disease and Cause of Death-7 (KCD-7), which is based on the ICD-10 (International Classification of Diseases-10) but modified for South Korea. The primary outcome was the incidence of CAA after the diagnosis of KD. The secondary outcomes included all cardiovascular complications, including CAA, coronary stenosis, angina pectoris, atherosclerosis of the arteries of the extremities, atrial fibrillation and flutter, other coronary heart diseases, acute myocardial infarction, thromboangiitis obliterans (Buerger’s disease), unstable angina, cardiovascular sequelae, myocarditis, right bundle branch block, heart disease, heart failure, cardiomyopathy, pericarditis, other acute pericarditis, other specified heart blocks, chronic constrictive pericarditis, supraventricular tachycardia, peripheral vascular disease, ventricular premature depolarization, rheumatic tricuspid stenosis, and nonrheumatic mitral valve insufficiency (Table S1).

### 2.3. Follow-Up

Patients were censored when diagnosed with CAA or cardiovascular complications, or for patients who were not censored, we terminated the follow-up on December 31 each year, whichever came first.

### 2.4. Statistical Analysis

In this study, we compared the distribution of basic patient characteristics between the two treatment groups (the IVIg group and the CS group). Categorical variables were analyzed using the chi-square test. Propensity scores, based on the patient characteristics (age and sex), were calculated using inverse probability of treatment weighting (IPTW) and applied in binary logistic regression. The incidence of CAAs and other cardiovascular complications was calculated. A Cox regression model was used to estimate the hazard ratios (HRs) for the study outcomes, comparing the two groups and their corresponding 95% CIs with adjustments for the propensity scores. All of the statistical analyses were conducted using SAS 9.4 (SAS Institute Inc., Cary, NC, USA), and a *p*-value of less than 0.05 was considered statistically significant.

## 3. Results

### 3.1. The Study Populations

**Figure 1:**
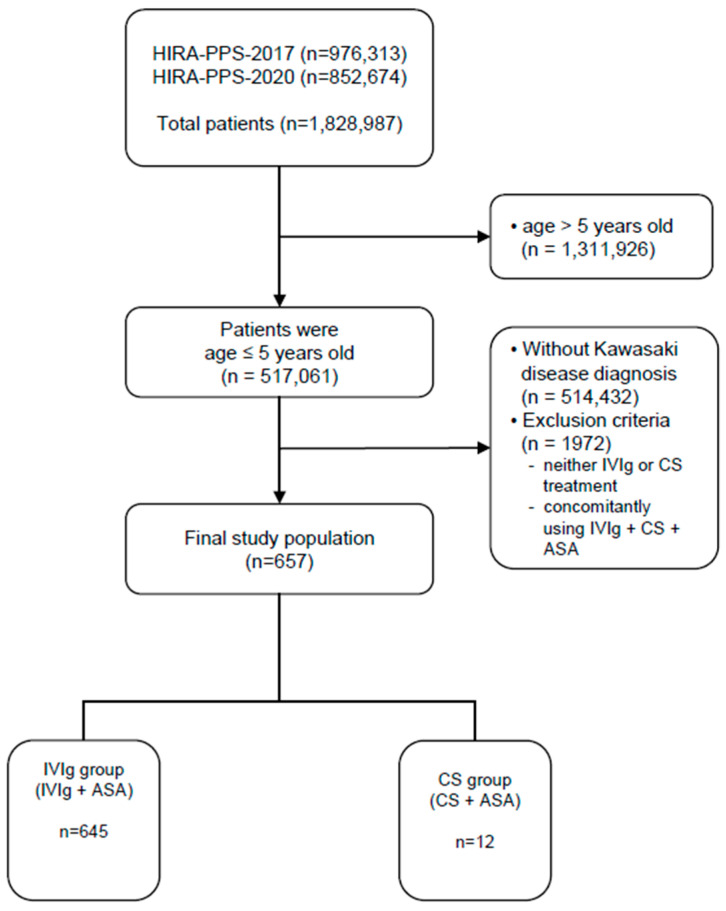
Flow diagram for study subject inclusion. IVIg, intravenous immunoglobulin; CSs, corticosteroids; ASA, acetylsalicylic acid.

A total of 1,828,987 patients were reviewed in the 2017 and 2020 datasets, of whom 517,061 patients were 5 years of age or younger. Of these, 2629 patients were diagnosed with KD, and a total of 657 patients were included in the final analysis, excluding patients to whom the exclusion criteria applied. No patient had been diagnosed with cardiovascular disease, including CAA, before being diagnosed with KD (Figure 1).

Of these, 645 patients received IVIg along with ASA, and only 12 patients received ASA and steroids. In both groups, boys were more prevalent than girls, and patients aged equal to or younger than 2 years were more prevalent. As a result of the propensity score weighting, the number of people in the IVIg group was 657, and the number in the CS group was 658. Additionally, approximately 70% of the KD patients were younger than 2 years of age, and approximately 55% were boys (Table 1).

### 3.2. Study Outcomes

Before the IPTW adjustment, CAA occurred in 12 out of 645 patients (1.86%) in the IVIG group and 1 out of 12 patients (8.33%) in the CS group, with no statistically significant difference observed. After the IPTW adjustment, CAA occurred in 12 out of 647 patients (1.86%) in the IVIG group and 44 out of 658 patients (6.72%) in the CS group. The incidence of CAAs was significantly higher in the CS group (1.86% vs. 6.72%), with an HR of 3.70 (95% CI: 1.96–6.97, *p* < 0.0001), indicating a statistically significant difference (Table 2).

For all cardiovascular complications, including CAAs, the difference between the two groups became more pronounced after the IPTW adjustment. Before the IPTW adjustment, cardiovascular complications occurred in 39 out of 645 patients (6.05%) in the IVIG group and 2 out of 12 patients (16.67%) in the CS group, with no statistically significant difference. However, after the IPTW adjustment, cardiovascular complications occurred in 40 out of 647 patients (6.05%) in the IVIG group and 115 out of 658 patients (17.48%) in the CS group. The CS group demonstrated a significantly higher incidence of cardiovascular complications (6.05% vs. 17.48%), with an HR of 2.87 (95% CI: 1.96–6.97, *p* < 0.0001), reflecting a statistically significant difference (Table 2).

The median follow-up period was 213 days (IQR: 110–307) in the IVIG group and 202.5 days (IQR: 121–296) in the CS group, with no statistically significant difference between the two groups.

## 4. Discussion

KD is an acute type of pediatric vasculitis, and IVIg is the first-line treatment recommended by the international guidelines. However, IVIg shortages and contraindications have led to increasing interest in CSs as a potential alternative treatment. This study aimed to assess whether CSs could serve as a substitute for IVIg by comparing the incidence of CAAs and other cardiovascular complications in KD patients treated with IVIg versus CSs using a nationwide claim database. Given the small sample size of the CS group, we applied IPTW to balance the baseline characteristics between the groups and obtain more reliable estimates.

Our findings demonstrated that after the IPTW adjustment, the incidence of CAAs was significantly higher in the CS group (6.72%) compared to that in the IVIg group (1.86%), with an HR of 3.70 (95% CI: 1.96–6.97, *p* < 0.0001). These results indicate that CS monotherapy is associated with a higher risk of cardiovascular complications and does not provide the same protective effect as IVIg. Therefore, while CSs may be beneficial as an adjunct therapy in IVIg-resistant or high-risk patients, they should not replace IVIg as the standard treatment for acute KD. These findings reinforce the current clinical guidelines that recommend IVIg as the primary treatment strategy for KD.

This followed the same pattern in previous studies, where the prevalence of CAAs decreased to less than 5% when high-dose IVIG and ASA were used in the initial treatment [6]. However, the absolute incidence of CAAs observed here was lower than that in previous studies. This is because our study excluded patients who were resistant to IVIg, for example, an IVIg + ASA + CS treatment group or a cyclosporine treatment group, and the absolute incidence of cardiovascular complications such as CAAs was lower than that in previous studies. A survey study conducted at 206 hospitals in Korea from 2015 to 2017 reported that approximately 95% of patients with acute KD used IVIg and that approximately 1.7% of them developed CAAs. Although in our study we cannot confirm the proportion of patients who used IVIg among all patients with KD and the proportion of patients who developed cardiovascular complications such as CAAs among all KD patients, these results are similar to those in our study, where IVIg was used in most patients, and the incidence of CAAs was less than 2% [28].

KD is a disease with significant regional differences in prevalence in East Asia [29]. According to an epidemiological study in Japan in 2018, 359 cases per 100,000 occurred in children under 5 years of age, and the highest rate occurred in boys aged 9 to 11 months, at 572 per 100,000 [30,31]. In South Korea, the incidence rate for KD was approximately 197 per 100,000 from 2015 to 2017 [28]. In comparison, in the United States, approximately 18–19 cases per 100,000 occurred in children under 5 years of age [32], and compared to East Asia, the incidence rate could be seen to decrease to about one-tenth. Therefore, interest in the treatment of KD patients is high in East Asia, including Korea, and it is very important to establish a basis for alternative treatments, especially when the supply or use of IVIg is limited.

In a previous study, the fact that the CS group did not show a significant difference in the incidence of CAAs compared to that in the IVIg group may be a result of an insufficient number of patients [19]. A previous study compared the incidence of CALs in IVIg and CS use, including 20 patients in each group [19]. In our study, before applying IPTW, the CS group had a small number of 12 patients, and at that time, there was no statistically significant difference in the clinical outcomes between the two groups. However, when the propensity score was applied and each group was statistically weighted to approximately 650 people, the incidence of CAAs or cardiovascular complications, including CAAs, became statistically significant. Given the small sample size of the CS group in the unweighted analysis, the statistical power was low. However, after the IPTW adjustment, the statistical power improved significantly, making the results more reliable. Therefore, according to this study, it can be confirmed that administering only CSs as an IVIg replacement drug in KD patients increases the risk of complications such as CAAs, and therefore, in situations where IVIg cannot be administered, it is necessary to select another appropriate drug or additional close cardiac monitoring, such as echocardiography.

In groups at high-risk of IVIg resistance, the additional use of glucocorticoids or cyclosporine is recommended [18,33]. Ultimately, these treatments cannot be the recommended therapy when IVIg use is limited. In a randomized controlled trial conducted in Japan, when ulinastatin was used as the initial treatment in KD patients, it was not effective in preventing CALs compared to the outcomes in the group using IVIg [34]. A retrospective study using ulinastatin in addition to IVIg showed that it reduced the incidence of CALs, but it was not a replacement for IVIg [35]. Similar to our study results, evidence for another therapeutic regimen that could be used as an alternative to IVIg in KD patients has not been established in existing studies.

This study provides important insights into the comparative effectiveness of IVIg and CSs in the treatment of KD, particularly in situations where IVIg administration may be limited. Our findings suggest that the combination of CSs and ASA is associated with a significantly higher risk of CAAs and cardiovascular complications compared to that with IVIg and ASA treatment. These results emphasize that while CSs are effective as an adjunctive therapy in IVIg-resistant or high-risk KD patients, their use as a standalone treatment should be approached with caution. In KD patients, IVIg shows a dose-dependent effect. Therefore, the highest dose for which evidence has been established in clinical trials is used in KD patients [21].

Given increasing global concerns over IVIg shortages, future research should carefully evaluate potential alternative strategies while considering the well-established dose–response relationship for IVIg. While adjunctive therapies, such as CSs or other immunomodulatory agents like cyclosporine, have been explored, the current evidence remains insufficient to support any modification of the standard high-dose IVIg regimen. Further clinical investigations are necessary to assess the safety and efficacy of alternative approaches in IVIg-limited settings, ensuring that the treatment recommendations remain aligned with established clinical guidelines

This study has several limitations. Since the data were obtained from a claim database, detailed clinical parameters such as inflammatory markers, fever duration, and echocardiographic findings were not available, which may have influenced the treatment decisions and outcomes. Furthermore, the CS group was relatively small before the IPTW adjustment, limiting the generalizability of our findings. Additionally, given the observational nature of this study, residual confounding cannot be completely ruled out, and prospective randomized controlled trials are needed to validate our results. Nevertheless, this study has several strengths. First, it is one of the few large-scale studies to utilize a nationwide healthcare claim database to compare IVIg and CS therapy in KD. The use of IPTW allowed for a more balanced comparison between the treatment groups, minimizing potential confounding factors and presenting statistical power. Additionally, by including a broad range of cardiovascular complications beyond CAAs, our study provides a comprehensive assessment of the potential risks associated with CS and ASA treatment in KD patients.

## 5. Conclusions

This study suggests that CS and ASA treatment is associated with an increased risk of CAAs and cardiovascular complications compared to that in IVIg therapy in KD patients. While IVIg remains the standard of care, concerns about its availability warrant further investigation into alternative treatment strategies. Future research using clinical records would allow for a more comprehensive evaluation of the impact of inflammatory markers, disease severity indicators, and imaging findings on the treatment outcomes. Until robust evidence supports alternative regimens, clinicians should exercise caution in managing KD patients in IVIg-limited settings, ensuring close cardiovascular monitoring and individualized treatment approaches.

## Figures and Tables

**Table 1:** Patient characteristics before and after weighting by propensity score.

Patient Characteristics	Before Weighting by Propensity Score			After Weighting by Propensity Score		
	IVIg Group (*n* = 645), *n* (%)	CS Group (*n* = 12), *n* (%)	*p*-Value	IVIg Group (*n* = 657), *n* (%)	CS Group (*n* = 658), *n* (%)	*p*-Value
Age			0.6783			0.8400
≤2 years	465 (72.1)	8 (66.7)		473 (72.0)	477 (72.5)	
>2 and ≤5 years	180 (27.9)	4 (33.3)		184 (28.0)	181 (27.5)	
Sex			0.9208			0.4854
Male	367 (56.9)	7 (58.3)		374 (56.9)	362 (55.0)	
Female	278 (43.1)	5 (41.7)		283 (43.1)	296 (45.0)	

IVIg, intravenous immunoglobulin G; CSs, corticosteroids.

**Table 2:** Comparison of clinical outcomes between IVIg group and CS group before and after weighting by propensity score.

Patient Characteristics	Before Weighting by Propensity Score				After Weighting by Propensity Score			
	IVIg Group (*n* = 645), *n* (%)	CS Group (*n* = 12), *n* (%)	HR (95% CI)	*p*-Value	IVIg Group (*n* = 657), *n* (%)	CS Group (*n* = 658), *n* (%)	HR (95% CI)	*p*-Value
CAA								
no	633 (98.1)	11 (91.7)	Reference		645 (98.2)	614 (93.3)	Reference	
yes	12 (1.9)	1 (8.3)	4.74 (0.62–36.48)	0.1348	12 (1.8)	44 (6.7)	3.70 (1.96–6.97)	<0.001
CV complications								
no	606 (94.0)	10 (83.3)	Reference		617 (93.9)	543 (82.5)	Reference	
yes	39 (6.0)	2 (16.7)	2.88 (0.67–11.94)	0.1442	40 (6.1)	115 (17.5)	2.87 (1.99–4.13)	<0.001

IVIg, intravenous immunoglobulin G; CSs, corticosteroids; HR, hazard ratio; CI, confidence interval; CAA, coronary artery aneurysm; CV, cardiovascular.

## Data Availability

The data are unavailable due to the privacy and ethical restrictions of HIRA.

## Supplementary Materials

The following supporting information can be downloaded at https://www.mdpi.com/article/10.3390/jcm14062012/s1. Table S1: Diseases and KCD-7 codes used in the analysis.

## Abbreviations

The following abbreviations are used in this manuscript:

ATC	Anatomical Therapeutic Chemical
ASA	acetylsalicylic acid
CAA	coronary artery aneurysm
CI	confidence interval
CS	corticosteroid
HR	hazard ratio
ICD	International Classification of Diseases
IPTW	inverse probability of treatment weighting
IQR	interquartile range
IVIg	intravenous immunoglobulin
KCD	Korean Standard Classification of Diseases and Causes of Death
KD	Kawasaki disease
NHI	National Health Insurance
