# Supplementary material for: The Comparative Effectiveness of Intravenous Immunoglobulin and Corticosteroids in Kawasaki Disease: A Nationwide Claim Data Analysis
Source: J Clin Med. 2025 Mar 16;14(6):2012. doi: 10.3390/jcm14062012 (PMC11943333; doi:10.3390/jcm14062012)
Supplement: Supplementary file 1 [file jcm-14-02012-s001.zip › jcm-3517029-supplementary.pdf]

Supplementary Material

Table S1. Diseases and KCD-7 codes used in analysis.

Diseases	KCD-7 code
Mucocutaneous lymph node syndrome [Kawasaki Disease]	M30.3
Coronary artery aneurysm	I25.4
Coronary stenosis	I25.1
Angina pectoris unspecified	I20.9
Atherosclerosis of arteries of extremities unspecified	I70.29
Atrial fibrillation and flutter	I48
Other coronary heart disease	I25.1
Acute myocardial infarction	I24.0
Thromboangiitis obliterans [Buerger's disease]	I73.1
Unstable angina	I20.0
Cardiovascular Sequelae	I40.9
Myocarditis, unspecified	I51.6
Right bundle-branch block	I45.1
Heart disease, unspecified	I51.9
Heart failure, unspecified	I50.9
Cardiomyopathy	I51.4
Pericarditis, unspecified	I31.9
Other acute pericarditis	I30.8
Other specified heart block	I45.5
Chronic constrictive pericarditis	I31.3
Supraventricular tachycardia	I47.1
Peripheral vascular disease, unspecified	I73.9
Ventricular premature depolarization	I49.3
Rheumatic tricuspid stenosis	I07.1
Nonrheumatic mitral (valve) insufficiency	I34.0

KCD-7, Korean standard classification of disease and cause of death-7
